# Investigation of molecular immune regulation mechanisms and therapeutic target prediction in pancreatic cancer based on bioinformatics and genetics

**DOI:** 10.1016/j.clinsp.2026.100952

**Published:** 2026-04-18

**Authors:** Changjun Dong, Jing Guan, Linhuan Dong, Yunlin Yu, Xiangwei Zhang, Zheng Li, Xianlin Zhang, Chunlin Mo

**Affiliations:** a Department of General Surgery II, Foshan Sanshui District People’s Hospital, Foshan, Guangdong, China; b Department of General Surgery, Affiliated Renhe Hospital of China Three Gorges University, Yichang, China; c Department of Critical Care Medicine, Foshan Sanshui District People’s Hospital, Foshan, Guangdong, China; d Hubei Key Laboratory of Tumor Microenvironment and Immunotherapy, China Three Gorges University, Yichang, China

**Keywords:** Pancreatic cancer, CLIC3, MST1R, Immune microenvironment, Bioinformatics, Clinical translation

## Abstract

**Objective:**

Pancreatic Cancer (PC) is a highly aggressive malignancy with a dismal prognosis, primarily due to late-stage diagnosis and limited therapeutic options. This study aimed to identify potential biomarkers involved in PC progression and immune microenvironment modulation.

**Methods:**

RNA sequencing data from The Cancer Genome Atlas (TCGA) and Genotype-Tissue Expression (GTEx) databases were integrated to identify differentially expressed genes. Mendelian Randomization (MR) analysis was conducted to assess causal associations between candidate genes and PC risk. Immune cell infiltration was evaluated using the CIBERSORT algorithm, and mediation analysis explored the role of immune cells in PC progression. Validation was performed using RT-qPCR and immunohistochemistry in clinical tissue samples.

**Results:**

CLIC3 and MST1R were significantly overexpressed in PC tissues and associated with advanced tumor stages. MR analysis confirmed their causal relationship with PC, with Odds Ratios of 2.36 (95% CI: 1.58–3.51) for CLIC3 and 1.30 (95% CI: 1.06–1.60) for MST1R. High expression of these genes correlated with increased M0 macrophages and decreased CD8⁺ T cells, CD4⁺ T-cells, and naïve B-cells, suggesting immune dysregulation. Mediation analysis emphasized the pivotal role of CD8⁺ cytotoxic T-cells in PC progression. Moreover, CLIC3 and MST1R were closely associated with immune checkpoint molecules CD276 and NT5E.

**Conclusions:**

CLIC3 and MST1R are overexpressed and causally implicated in pancreatic cancer development and immune modulation. They represent promising biomarkers and potential therapeutic targets for precision immunotherapy in PC.

## Highlights

• CLIC3 and MST1R are overexpressed and linked to pancreatic cancer progression.
• Mendelian randomization confirms a causal role of CLIC3 and MST1R in PC.
• High expression of both genes alters immune infiltration, especially CD8+ T-cells.
• CLIC3 and MST1R correlate with poor prognosis in pancreatic cancer patients.
• Both genes are potential targets for precision and immune-based PC therapies.

## Introduction

PC is one of the deadliest malignancies of the digestive system, often diagnosed at advanced stages, resulting in missed surgical opportunities and significantly increasing the challenges of early diagnosis and treatment. The pathogenesis and progression of PC are characterized by high complexity, with interconnected biological pathways, making its treatment strategies and drug development a highly challenging endeavor.^1^^,^^2^ With the rise of precision medicine, researchers have begun to focus on the molecular functions and genetic information of PC, leveraging multi-omics data to tailor more precise treatment strategies for patients. A comprehensive investigation into the molecular mechanisms of PC, particularly the role of the immune microenvironment and drug targets, will provide crucial support for precision treatment approaches. Bioinformatics, as a core tool for analyzing large-scale biological data, has emerged as a pivotal driving force in cancer research.^3^^,^^4^ By utilizing high-throughput data and machine learning algorithms, bioinformatics can uncover potential immune molecular mechanisms and novel therapeutic targets in PC, thereby offering valuable insights for clinical research.^5^^,^^6^ Currently, several genes have been identified as risk factors for PC, involved in processes such as tumor proliferation and metastasis, modulating immune mechanisms, and potentially serving as novel drug targets.^7^, ^8^, ^9^, ^10^

This study employs bioinformatics analysis and Mendelian Randomization (MR) to identify a positive causal relationship between Chloride Channel protein-3 (CLIC3) and Macrophage Stimulating Receptor-1 (MST1R) in PC, which are closely associated with alterations in the immune microenvironment and may serve as potential therapeutic targets. As a member of the intracellular chloride ion channel family, CLIC3 has been shown to be associated with various tumors, with low expression linked to poor prognosis in gastric cancer, while high expression is correlated with unfavorable clinical-pathological features in bladder cancer and other malignancies.^11^, ^12^, ^13^ MST1R, a member of the C-Met family, is involved in the initiation and progression of various cancers and acts as a potential oncogene by enhancing tumor cell proliferation and invasion.^14^, ^15^, ^16^, ^17^ Studies have shown that high expression of MST1R in PC is closely associated with macrophage infiltration, exerting a profound impact on the onset and progression of the disease.^18^ Although previous studies have highlighted the roles of CLIC3 and MST1R in PC, their genetic causal relationships, molecular immune mechanisms, and predictions as drug targets remain insufficiently validated. Given the immune evasion mechanisms of PC, conventional surgical treatments and single-target therapies can no longer meet the clinical needs. Despite progress with PARP inhibitors and immune checkpoint inhibitors, drug resistance remains a major challenge.^19^, ^20^, ^21^ Multi-target drugs, such as cabozantinib, have shown promising results in clinical studies, particularly in advanced renal and thyroid cancers.^22^^,^^23^ Therefore, exploring the molecular immune mechanisms of PC and identifying novel drug targets is crucial for improving therapeutic outcomes.

This study employs transcriptomic data to identify key genes and utilizes MR to validate their causal relationship with PC, combining multi-omics analysis to explore their molecular immune mechanisms and further assess the binding potential of these genes with novel drug targets. For experimental validation, the authors will analyze PC cell lines and clinical samples using RT-qPCR and immunohistochemistry to assess the expression differences of key genes and their roles in the pathogenesis of PC. This study not only advances our understanding of the molecular immune regulatory mechanisms in PC but also provides a novel theoretical foundation and practical direction for future clinical translational research.

## Materials and methods

### Data collection and processing

A total of 182 PC samples, including 178 tumor samples and 4 normal samples, were retrieved from the TCGA dataset (https://www.cancer.gov/). Additionally, 167 normal pancreatic samples were collected from the GTEx dataset (https://xenabrowser.net/). After batch effect correction, the mRNA expression data from all samples were merged into a single dataset for subsequent analysis.

### Differential expression analysis

Use the “limma”, “DEseq2”, “edgeR” packages and the Wilcoxon rank sum test from R 4.3.3 version (https://www.R-project.org) to perform differential expression analysis. The screening conditions are |logFC| > 1, adjusted p-value < 0.05, and FDR multiple hypothesis testing correction is used to obtain up-regulated and down-regulated sets of differential genes.

### Weighted gene co-expression network analysis

The samples were divided into tumor and normal groups, with outlier samples removed through clustering. To stabilize the correlation between different nodes in the network, the “WGCNA” package was used to select an appropriate soft threshold parameter (power). Based on this threshold, a weighted network was calculated, and a scale-free network was constructed. Key module genes in the tumor group were then identified.

### Two-sample mendelian randomization analysis

The genes identified through differential analysis were intersected with the key module genes from the WGCNA.eQTL data from the GTEx V8 database (https://gtexportal.org/home/) were selected as the exposure variable, while Genome-Wide Association Study (GWAS) data from the FinnGen release 10 (https://www.finngen.fi/en), comprising 1626 PC cases and 314,193 European ancestry controls, were used as the outcome variable.MR analysis was performed using the “TwoSampleMR” package. Instrumental Variables (SNPs) were selected based on the P-value of the SNPs (p < 1 × 10^–5^), with the following three assumptions for the selection of these instrumental variables: 1) The SNP must be strongly associated with the exposure; 2) The SNP affects the outcome only through the exposure; 3) The SNP is independent of confounders. The Linkage Disequilibrium (LD) parameter R^2^ was set to 0.1, and the genetic distance was set to 100 kb to ensure the independence of SNPs and to remove potential LD effects. Only SNPs with an *F*-statistic greater than 10 were considered, and p-values were adjusted using the Bonferroni method to ensure that the SNPs were strongly associated with the exposure, influenced the outcome solely through the exposure, and were not confounded by other factors. Heterogeneity was assessed using Cochran's *Q* statistic and the MR-Egger method, while horizontal pleiotropy was corrected using the MR-PRESSO method. The influence of each SNP on the overall results was evaluated using the leave-one-out method.

### Survival analysis

Univariate Cox regression analysis was performed on genes with a p-value < 0.05 from the MR analysis using the “survival” and “glmnet” packages, and the results were visualized. Genes consistent with the MR results were selected for multivariate Cox regression analysis to evaluate their role as independent risk factors.

### Clinical correlation analysis

Clinical information for PC patients was obtained from the Cancer Genome Atlas (TCGA) database. Data extraction and organization were performed using Perl (https://www.perl.org/). The study analyzed several clinical variables, including tumor grade, alcohol history, smoking history, and history of chronic pancreatitis. Regarding tumor grade, patients were classified into G1 (n = 29), G2 (n = 96), G3 (n = 51), and G4 (n = 2). In terms of tumor stage, 19 patients were classified as Stage I (n = 19), 30 as Stage IIA (n = 30), 120 as Stage IIB (n = 120), and 9 as Stage III/IV (n = 9). For alcohol history, 50 patients were categorized as alcohol users (n = 50), while 73 were classified as non-users (n = 73). Smoking history was divided into smokers (n = 105) and non-smokers (n = 18). Lymph node count was categorized as more than 20 lymph nodes (n = 36) and 20 or fewer lymph nodes (n = 87). Additionally, 10 patients had a history of chronic pancreatitis (n = 10), while 113 patients did not (n = 113). These clinical variables were analyzed using the “ggpubr”, “limma”, and “ComplexHeatmap” R packages. Non-parametric rank-sum tests were performed for group comparisons, and the results were visualized to provide a comprehensive analysis of the clinical characteristics and stratification of the patient cohort.

### Immune infiltration analysis

Immune cell infiltration in tumor tissues was analyzed using the CIBERSORT method (Cell-type Identification by Estimating Relative Subsets of RNA Transcripts), which is based on linear Support Vector Regression (SVR).^24^ Gene expression data were processed using the “limma” package, with negative values and missing data removed.^25^ The relative abundance of different immune cell types in PC tissues was calculated using the “CIBERSORT.R” script, and differences in immune cell infiltration were compared between high and low expression groups of the target gene. Spearman correlation analysis was performed to evaluate the relationship between the target gene and immune cells. These analyses were performed to explore associations between gene expression levels and immune cell infiltration patterns and do not imply direct causal relationships.

### Consensus clustering and immune profiling based on CLIC3 and MST1R

Unsupervised consensus clustering was performed on CLIC3 and MST1R expression profiles using the Consensus Cluster Plus R package, determining the optimal number of clusters based on Cumulative Distribution Function (CDF) curves and consensus matrices. Immune cell compositions among the defined subgroups were estimated via CIBERSORT, and survival analyses were performed using Kaplan-Meier curves and log-rank tests.

### Immune cell-mediated MR analysis

Data for 731 immune phenotypes were obtained from the latest GWAS study of 3757 individuals of Sardinian ancestry, including 3575 cases and 3027 controls.^26^ Two-Step Mendelian Randomization (2SMR) analysis was performed using the “TwoSampleMR” package and the MR-PRESSO tool. First, eQTL data of the target gene were used as the exposure variable, with immune phenotypes as the outcome, to estimate the causal effect (beta1). Secondly, immune phenotypes were used as the exposure variable, with PC as the outcome, to calculate the causal effect (beta2). The mediating effect and its proportion were calculated to evaluate the role of immune cells in PC.

### Immune checkpoint blockade analysis

Based on the median expression level of target genes, patients were divided into high-expression and low-expression groups. Comparing the expression levels of the 12 common Pancreatic Cancer (PC) immune checkpoints ‒ PDCD1, CD274, PDCD1LG2, CD86, LAG3, HAVCR2, CD276, VTCN1, IDO1, TIGIT, CD47, and NT5E ‒ helps to further elucidate the potential regulatory mechanisms of these target genes in the PC immune microenvironment and their role relationships.

### Enrichment analysis

Gene Ontology (GO) and Kyoto Encyclopedia of Genes and Genomes (KEGG) functional enrichment analyses were performed on the intersecting genes using the “org.Hs.eg.db”, “clusterProfiler”, and “enrichplot” packages to assess the enrichment of the gene set in known functional pathways. The gene sets were then ranked according to the log2 fold change of the target genes, and Gene Set Enrichment Analysis (GSEA) was conducted to reveal the trend of pathway or biological process changes.

### Co-expression analysis and protein-protein interaction (PPI) network construction

Spearman correlation analysis was used to calculate the co-expression relationship between the target gene and other genes. A Protein-Protein Interaction (PPI) network was constructed using the STRING database (https://cn.string-db.org/). Network features were evaluated based on the degree of protein connectivity.

### Colocalization analysis

The eQTL data of the target genes and the GWAS data for PC were organized. The regions 500 kb upstream and downstream of the genes were selected, and missing columns, such as standard deviation and imputed Allele Frequency (EAF), were calculated to ensure inclusion of all SNPs. Colocalization analysis and visualization were performed using the “coloc” and “locuscomparer” packages. The prior probability for a shared causal variant between the target gene eQTL and pancreatic cancer GWAS signals (p12) was set to 1.0 × 10^–4^, which is a commonly used default value in colocalization analyses of complex traits, as recommended in previous methodological studies and the original coloc framework.^27^ The authors acknowledge that the choice of prior probability may influence posterior estimates, and therefore, the colocalization results should be interpreted in the context of this assumption.

### Tumor mutational burden (TMB) analysis

Mutation data for 178 PC patients were obtained from the TCGA database. Patients were categorized into high-expression and low-expression groups based on the expression levels of the target gene. Gene mutation rates in both groups were analyzed using the “maftools” package, and the top 30 mutated genes were displayed. Tumor Mutational Burden (TMB) for each sample was calculated and correlated with the expression levels of the target gene.

### Target drug discovery and molecular docking

Drugs or compounds associated with the target gene were retrieved from the DSigDB database (https://dsigdb.tanlab.org/DSigDBv1.0/). Corresponding small molecule structures were downloaded from the PubChem database (https://pubchem.ncbi.nlm.nih.gov/). The three-dimensional structure of the target gene's protein was obtained from the PDB database (https://www.rcsb.org/). Molecular docking was performed using the CB-Dock2 online platform (https://cadd.labshare.cn/cb-dock2/index.php). The binding energy between the drugs and the target gene was evaluated.^28^^,^^29^

### Validation of key gene expression by RT-qPCR

The hTERT-HPNE, PANC-1, BxPC-3, and AsPC-1 cell lines were cultured using different media conditions. The hTERT-HPNE cells were cultured in high-glucose DMEM supplemented with 10% Fetal Bovine Serum (FBS) and 1% Penicillin-Streptomycin (P/S); PANC-1 cells were cultured in high-glucose DMEM with 10% FBS and 1% P/S; BxPC-3 and AsPC-1 cells were cultured in RPMI-1640 medium supplemented with 10% FBS and 1% P/S. All cells were cultured at 37 °C in a 5% CO_2_ humidified atmosphere, with media replaced every 2‒3 days. Subculturing was performed when cells reached 80%‒90% confluence. RT-qPCR was employed to assess the differential expression of the target gene across various cell lines. Primers were designed using Primer software and synthesized and validated by Wuhan Biological Engineering Company. ACTIN was selected as the reference gene, and qPCR amplification was performed under identical conditions for both the target gene and the reference gene.

### Immunohistochemistry (IHC) validation of key genes

Ten clinical pathological samples diagnosed as PC between 2014 and 2024 from the Department of General Surgery, Renhe Hospital, Three Gorges University, were selected for analysis. Paraffin-embedded sections of PC tissue and corresponding adjacent non-tumor tissue were used as normal controls for within-group comparison. The procedures followed included deparaffinization, dehydration, antigen retrieval, blocking of endogenous peroxidase activity, and incubation with primary and secondary antibodies. DAB staining was performed, followed by counterstaining, dehydration, and mounting of the tissue sections. The stained slides were examined using a light microscope and evaluated for staining intensity. Digital scanning was carried out with a SEVIR scanner, and ImageJ software was employed for the analysis of immunohistochemical staining images. The Average Optical Density (AOD) of PC tissues and adjacent non-tumor tissues was calculated. Group differences were analyzed using non-parametric statistical methods.

### Statistical analysis

Statistical analyses were conducted using R (version 4.3.3) and GraphPad Prism-8. Spearman's rank correlation coefficient was used for correlation analysis. Batch effect correction was applied to all datasets prior to merging to minimize potential systemic biases. Group comparisons were assessed using the Wilcoxon rank-sum test, and a p-value of < 0.05 was set as the threshold for statistical significance. This study is based on the analysis of publicly available datasets and follows the STROBE (Strengthening the Reporting of Observational Studies in Epidemiology) guidelines for reporting observational studies.

## Results

### Differential expression analysis

A comprehensive analysis was performed on 182 samples from the TCGA database (including 178 PC cases and 4 controls) and 167 normal pancreatic tissue samples from the GTEx database, followed by batch effect correction (Supplementary Fig. S1A‒B). The mRNA expression matrix was then filtered, and differential expression analysis was conducted using four methods. The intersection of results identified 961 upregulated genes and 257 downregulated genes (Supplementary Fig. S1C‒D) for subsequent analysis.

### Weighted gene co-expression network analysis

**Fig. 1:**
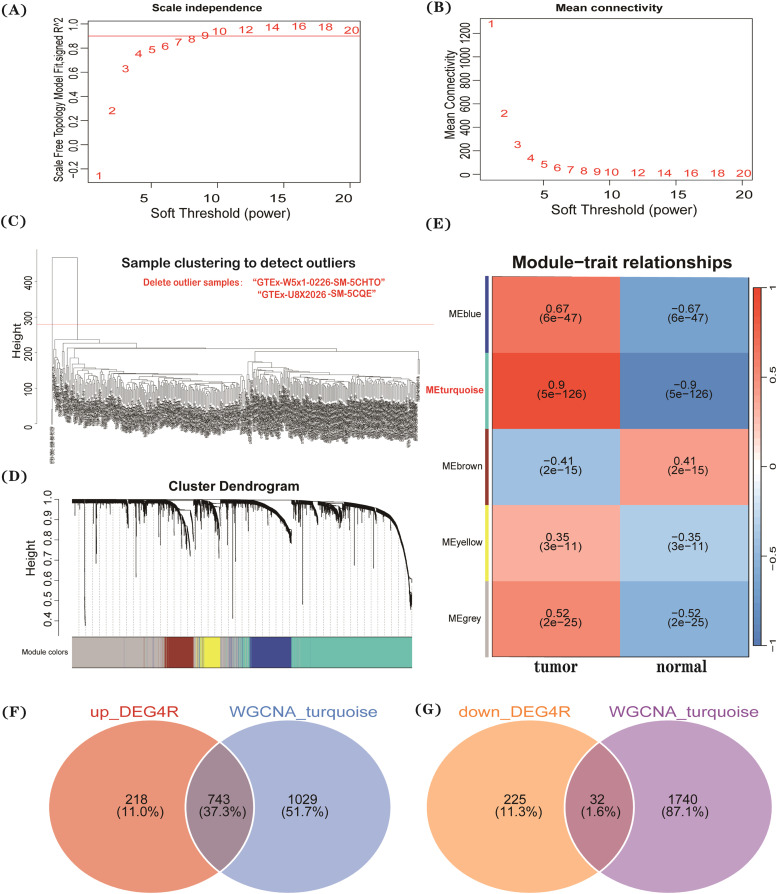
Samples were categorized into “tumor” and “normal” groups as external traits. The optimal soft-threshold power was determined (A‒B), followed by the exclusion of outlier samples (C). The Topological Overlap Matrix (TOM) was constructed, and clustering analysis was used to define relationship modules (D). A heatmap illustrating the correlation between each module and external traits, along with corresponding correlation coefficients and p-values, was generated (E).

The combined “TCGA+GTEx” dataset was categorized into tumor and normal groups, and two outlier samples were excluded based on a hierarchical clustering tree with a cut-height of 280. Using soft-threshold power analysis, a power exponent of 12 was selected to meet the scale-free network criteria. Following the generation of the Topological Overlap Matrix (TOM), the turquoise module demonstrated the strongest correlation with the tumor group (correlation coefficient = 0.9, p < 0.001) (Fig. 1A‒E). This module included 743 upregulated and 32 downregulated genes (Fig. 1F‒G), narrowing the scope of differential gene screening and facilitating the precise identification of key genes associated with PC.

### Two-sample MR analysis

**Fig. 2:**
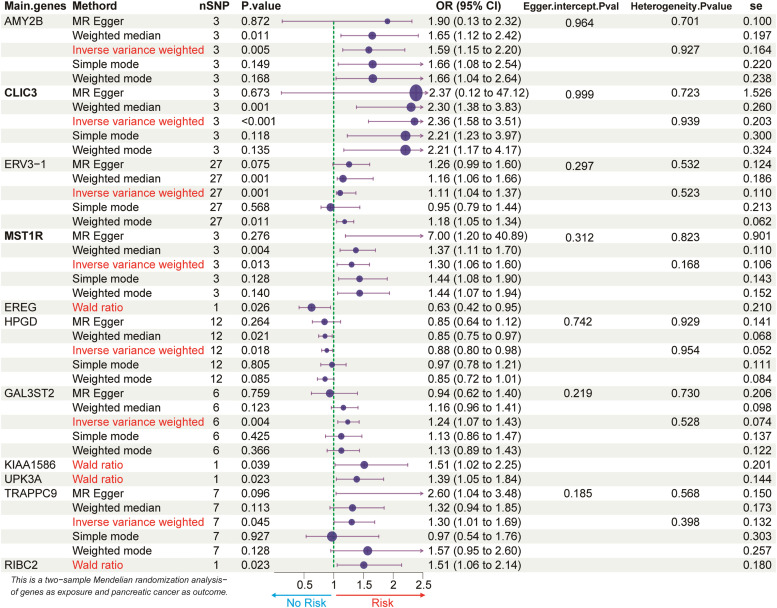
Two-sample MR analysis was conducted using the eQTL data of intersecting genes and GWAS data for PC, with only the top 11 genes showing the most significant p-values in the IVW method presented.

The authors conducted a two-sample MR analysis using the intersection genes from differential expression analysis and WGCNA, integrating eQTL data and GWAS data from the Finnish PC Genome Study to evaluate the causal relationships of these genes with PC. The IVW analysis identified 19 genes significantly associated with PC (Fig. 2). For instance, CLIC3 showed a positive association with PC (OR = 2.36, 95% CI 1.58–3.51, p < 0.001), while HPGD exhibited a negative association (OR = 0.88, 95% CI 0.80–0.98, p = 0.018). The Cochran’s *Q* test indicated no heterogeneity (p > 0.05), and the MR-PRESSO test detected no horizontal pleiotropy. Detailed p-values for the top 11 significant genes identified by IVW analysis are shown in Fig. 2.

### Survival analysis

**Fig. 3:**
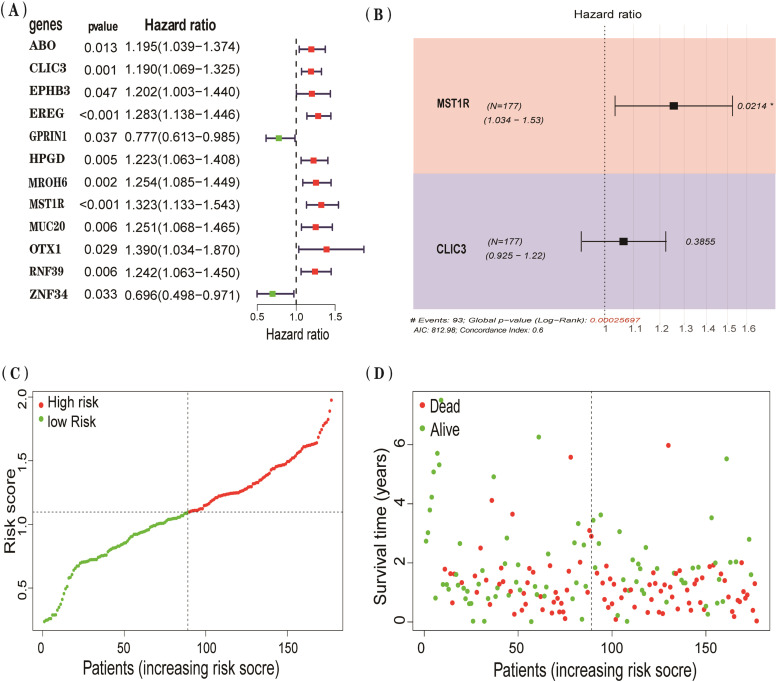
A forest plot of the univariate Cox regression analysis results is presented, showing only genes with p-values < 0.05 (A). A forest plot of the multivariate Cox regression analysis for key genes is displayed (B), along with the development of a prognostic risk score model (C‒D).

Among the 19 significant genes identified in the MR analysis, univariate Cox regression revealed that 12 genes, including ABO, CLIC3, EPHB3, and EREG, were significantly associated with PC survival (Fig. 3A). Genes with an Odds Ratio (OR) > 1 in MR analysis and a Hazard Ratio (HR) > 1 in Cox analysis were selected for further investigation. Both CLIC3 and MST1R met these criteria and were significantly associated with the risk of PC-related mortality (HR = 1.190, p < 0.01; HR = 1.323, p < 0.01). A subsequent multivariate Cox regression model indicated that MST1R is an independent risk factor for PC, while the effect of CLIC3 might be influenced by MST1R (Fig. 3B). Patients in the high-risk group exhibited significantly higher mortality rates compared to the low-risk group (Fig. 3C‒D).

### Clinical correlation analysis

To evaluate the correlation between CLIC3 and MST1R expression levels and clinical factors, PC patients were divided into high- and low-expression groups based on the median expression levels of each gene. Significant associations were observed between CLIC3 expression and tumor grade, history of alcohol consumption, and chronic pancreatitis (p < 0.05). Correlation analysis revealed significant differences in pathological grades between G1 and G2/G3 stages, as well as between G2 and G4 stages. Similarly, clinical stages exhibited significant differences between Stage I and Stage IIA/IIB. Risk factors such as alcohol consumption, smoking, chronic pancreatitis, and the presence of more than 20 peritumoral lymph nodes were also significantly associated (Fig. S2A‒E). These findings suggest that high CLIC3 expression influences PC proliferation and migration and is closely associated with risk factors such as smoking, alcohol consumption, and chronic pancreatitis. For MST1R, significant differences were observed in pathological grades (G1 vs. G2/G3; G2 vs. G4) and chronic pancreatitis status (Fig. S2F‒G). However, the correlation between MST1R and clinical factors was less pronounced and limited compared to CLIC3.

### Immune infiltration analysis

**Fig. 4:**
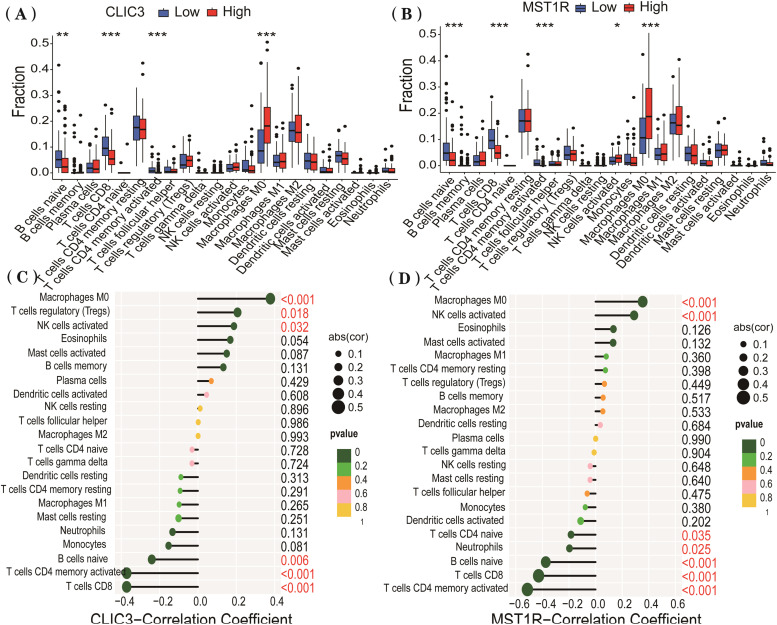
Immune cell infiltration patterns in the high- and low-expression groups of CLIC3 (A) and MST1R (B) in PC patients from the TCGA database. Correlation analysis of CLIC3 (C) and MST1R (D) with various immune cells is visualized.

The authors analyzed the immune infiltration patterns of CLIC3 and MST1R in 178 PC samples from the TCGA database. The results indicated that the high-CLIC3-expression group exhibited significantly increased infiltration of M0 macrophages, while infiltration of CD4+ T-cells, CD8+ T-cells, and B cells was markedly reduced (Fig. 4A). Furthermore, CLIC3 expression was positively correlated with M0 macrophages, regulatory T-cells (Tregs), and Natural Killer (NK) cells, but negatively correlated with CD4+ T-cells, CD8+ T-cells, and B-cells (Fig. 4C). Similarly, the high-MST1R-expression group showed elevated infiltration of M0 macrophages and NK cells (Fig. 4B), accompanied by decreased infiltration of CD4+ T-cells, CD8+ T-cells, and B-cells (Fig. 4D). These findings indicate that high expression levels of CLIC3 and MST1R are associated with increased infiltration of M0 macrophages and reduced abundance of CD4+ and CD8+ T-cells and B-cells in pancreatic cancer. Collectively, these associations suggest a potential link between CLIC3/MST1R expression and an immunosuppressive tumor microenvironment.

### Immune cell-mediated MR analysis

In the previous MR analysis, the total effect beta value of CLIC3 was calculated as 0.858 (Table 1). A two-step MR analysis was then performed, yielding beta1 and beta2 values of 0.317 and −0.514, respectively, for CD8br AC cells, and 0.507 and −0.123, respectively, for CD20 on IgD- CD27- cells. Since the indirect effect values were opposite in direction to the total effect, this suggests that these immune cells may play an inhibitory role in the causal relationship between the target gene and PC. After taking the absolute values of the indirect effects, the mediation proportions for CD8br AC and CD20 on IgD- CD27- cells were calculated as 18.99% and 7.27%, respectively. However, no statistically significant immune cells were identified as mediators for MST1R.

**Table 1:** Immune cell-mediated MR analysis.

Mediating factor	Total effect	Beta1	Beta2	|beta1*beta2|/ Total effect
CD8br AC	0.858	0.317	−0.514	18.99%
CD20 on IgD- CD27-	0.858	0.507	−0.123	7.27%

### Consensus clustering and immune profiling based on CLIC3 and MST1R

**Fig. 5:**
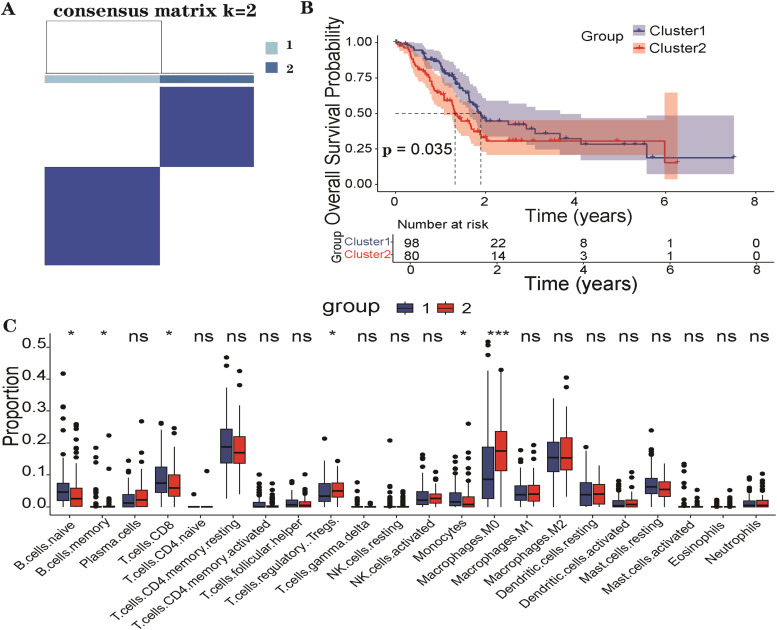
Consensus clustering based on CLIC3 and MST1R expression identifies two molecular subtypes of pancreatic cancer. Samples were stratified into Cluster 1 and Cluster 2 (A); Kaplan-Meier survival analysis revealed significantly poorer overall survival in Cluster 2 (p < 0.05) (B); Cluster 2 exhibited significantly higher infiltration of M0 macrophages and reduced CD8+ T-cell infiltration (p < 0.05) (C).

Consensus clustering stratified pancreatic cancer samples into two distinct clusters, Cluster 1 and Cluster 2 (Fig. 5A). Immune infiltration analysis revealed a significant enrichment of M0 macrophages and a reduction in CD8⁺ T-cells in Cluster 2 (p < 0.05) (Fig. 5C). Kaplan-Meier survival analysis indicated that patients in Cluster 2 had significantly worse overall survival compared with those in Cluster 1 (p < 0.05) (Fig. 5B).

### Immune checkpoint blockade analysis

The authors compared the expression of 12 immune regulatory drug targets between the high- and low-expression groups of CLIC3 and MST1R. The results indicated that LAG3, IDO1, and TIGIT were expressed at lower levels in the high-CLIC3-expression group, whereas PDCD1, PDCD1LG2, LAG3, HAVCR2, and TIGIT were expressed at lower levels in the high-MST1R-expression group. These findings suggest that these immune checkpoints may not be involved in the immune escape mechanisms driven by CLIC3 and MST1R. In contrast, the expression of CD276 and NT5E was elevated in both high-CLIC3 and high-MST1R expression groups, implying that they may serve as markers of immune escape and provide a theoretical basis for targeted therapy using combination immune checkpoint inhibitors and CLIC3/MST1R inhibitors (Fig. S3A‒B).

### Enrichment analysis

**Fig. 6:**
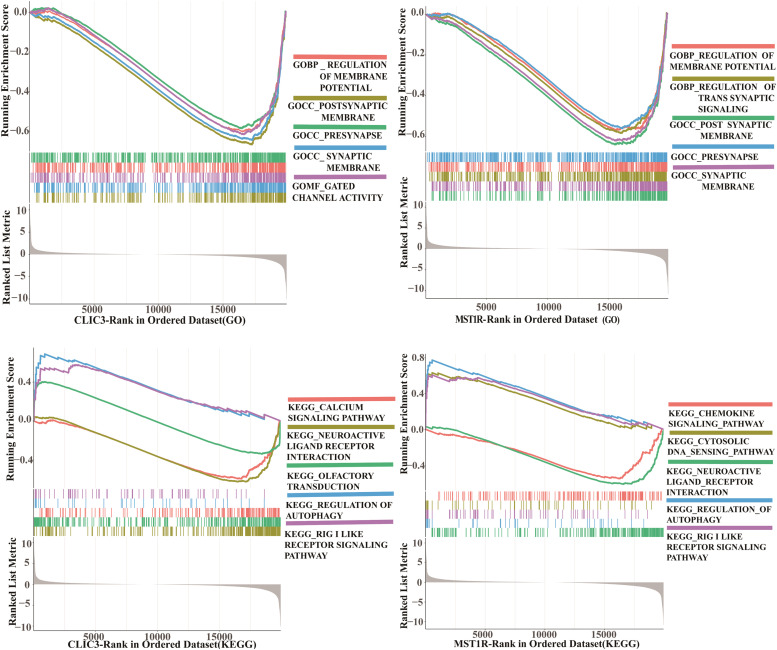
GO enrichment analysis for the single genes CLIC3 and MST1R (A‒B), and KEGG pathway enrichment analysis (C‒D).

To further explore the functions of CLIC3 and MST1R and their roles in specific biological processes, gene enrichment analyses were conducted for each gene. The Gene Ontology (GO) enrichment analysis indicated that both genes are primarily associated with synaptic structure and neuronal signal transduction, involving molecular mechanisms of synaptic transmission and transmembrane channel activities, including membrane potential regulation, synaptic signaling modulation, postsynaptic membranes, presynaptic sites, and gated channel activity (Fig. 6A‒B). Kyoto Encyclopedia of Genes and Genomes (KEGG) enrichment analysis revealed that the two genes are implicated in crucial biological processes such as immune response, autophagy regulation, neuronal signal transduction, intracellular DNA sensing, and chemokine signaling pathways, including RIG-I-like receptor signaling, autophagy regulation, neuroactive ligand-receptor interactions, cytoplasmic DNA sensing pathways, chemokine signaling pathways, olfactory transduction, and calcium signaling (Fig. 6C‒D). The enrichment analyses of CLIC3 and MST1R revealed similar biological functions, providing strong support for further investigation into their involvement in PC-related pathways and immune regulatory mechanisms, and highlighting their potential as therapeutic targets.

### Colocalization analysis and PPI

Under the specified prior probability setting (p12 = 1.0 × 10^–4^), Bayesian colocalization analysis indicated a high posterior probability (PP.H4 = 82.9%) for a shared genetic signal between CLIC3 and pancreatic cancer, surpassing the 55.7% observed for MST1R. These findings suggest that CLIC3 exhibits a stronger colocalization capacity and a more robust causal relationship with pancreatic cancer (Supplementary Fig. S4A‒B). The PPI network, derived from genes co-expressed with CLIC3, identified ITGB4, PLEC, PKP3, SNF, and JUP as pivotal players in the pathogenesis of pancreatic cancer (Supplementary Fig. S4D).

### Tumor mutational burden (TMB) analysis

The Tumor Mutational Burden (TMB) in the high-expression groups for CLIC3 and MST1R reached 98.81% and 100%, respectively, markedly exceeding the low-expression groups (79.22% and 78.21%). This observation suggests that tumors with elevated expression exhibit enhanced immunogenicity, producing a greater array of non-self-antigens that more effectively stimulate the immune system (Supplementary Fig. S5D‒G). Further analysis revealed a positive correlation between the expression levels of CLIC3 and MST1R and TMB scores, underscoring their potential role in tumor immunogenicity (Supplementary Fig. S5A‒B).

### Target drug discovery and molecular docking

Through candidate drug screening via the DSigDB database, the authors identified a range of targeted therapies, antitumor kinase inhibitors, and antibiotics linked to the target genes. Molecular docking analysis revealed that Cabozantinib, Bosutinib, and ILORASERTIB demonstrated strong hydrogen bonding interactions with MST1R, with Cabozantinib achieving the most favorable binding energy of −8.7. Similarly, within the CLIC3 crystal structure, IBMX, ampicillin, and HCtoxin established stable hydrogen bonds, with HCtoxin presenting the lowest binding energy at −7.5, underscoring its superior binding stability (Table 2, Supplementary Fig. S6).

**Table 2:** Predicted drugs and hydrogen bond energy.

Drugs Name	Target	Hydrogen Bond Binding Energy (kcal/moL)
Cabozantinib	MST1R	−8.7
Bosutinib	MST1R	−7.9
ILORASERTIB	MST1R	−8.1
IBMX	CLIC3	−6.8
Ampicillin	CLIC3	−6.3
HCtoxin	CLIC3	−7.5

### Validation of key gene expression by RT-qPCR

**Fig. 7:**
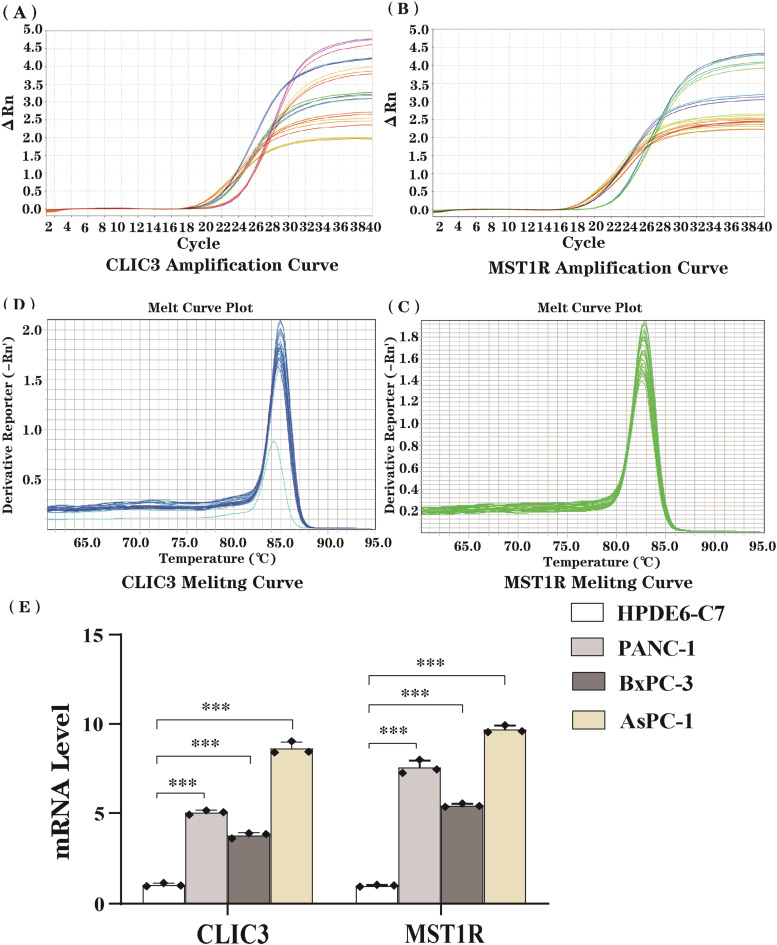
Real-time amplification results for CLIC3 and MST1R across different samples (A‒B) show an “S”-shaped curve, indicating high amplification efficiency. Melting curves for CLIC3 and MST1R demonstrate single melting peaks (C‒D). Bar charts illustrate mRNA expression levels of CLIC3 and MST1R in various cell lines (HPDE6-C7, PANC-1, BxPC-3, and AsPC-1) (E), showing significant differences in gene expression levels across cell lines (***p < 0.001).

To gain deeper insights into the expression profiles of CLIC3 and MST1R in PC, the authors employed RT-qPCR to analyze their mRNA levels in normal pancreatic epithelial cells (HPDE6-C7) and three PC cell lines (PANC-1, BxPC-3, and AsPC-1). Amplification curves for both genes displayed consistent exponential growth across all samples, indicative of excellent amplification efficiency (Fig. 7A‒B). Melting curve analysis revealed sharp, single peaks for both genes (Fig. 7C‒D), confirming the high specificity of the primers and eliminating potential interference from nonspecific amplification or primer dimer formation. RT-qPCR results demonstrated a pronounced upregulation of CLIC3 and MST1R mRNA levels in all PC cell lines relative to the normal cell line, with statistically significant differences (p < 0.001, Fig. 7E). CLIC3 expression reached its nadir in HPDE6-C7 and peaked in AsPC-1, exhibiting a nearly 10-fold increase. Similarly, MST1R expression was most pronounced in AsPC-1, with intermediate levels observed in PANC-1 and BxPC-3. Collectively (Fig. 7E), these findings highlight a potential link between the elevated expression of CLIC3 and MST1R and the tumorigenesis and progression of PC.

### IHC validation of key genes

**Fig. 8:**
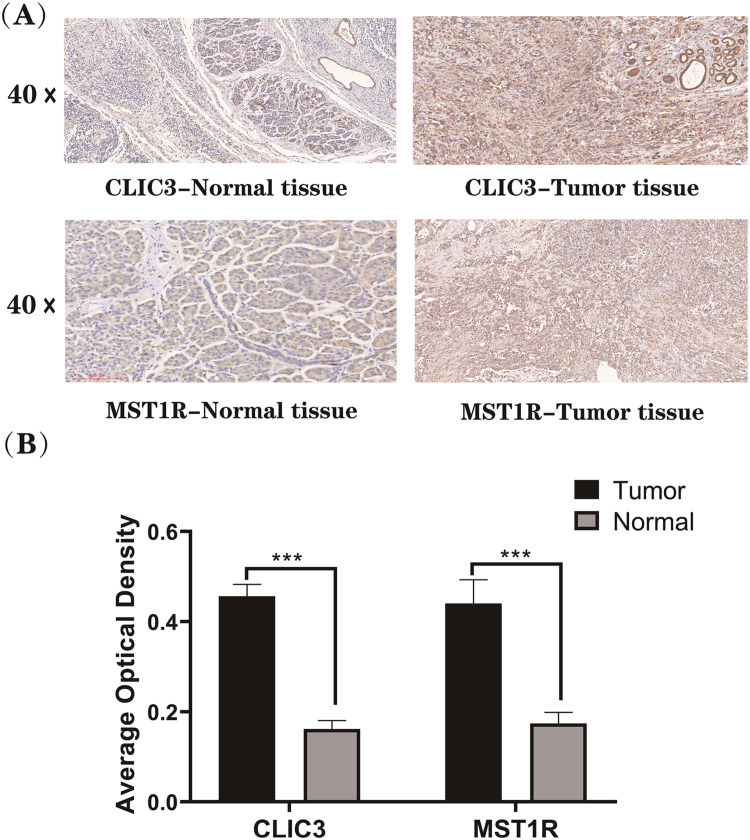
Immunohistochemical staining of CLIC3 and MST1R in normal pancreatic tissues and pancreatic cancer tissues (A), along with the differences in AOD values (*** indicates p < 0.001).

In Immunohistochemistry (IHC) analysis, Average Optical Density (AOD) was employed as a quantitative metric for staining intensity to assess the expression levels of CLIC3 and MST1R in PC tissues and adjacent noncancerous tissues (Fig. 8A). The results revealed that staining intensity in PC tissues was significantly elevated compared to adjacent noncancerous tissues (p < 0.05) (Fig. 8B), further corroborating the proteomic evidence of CLIC3 and MST1R overexpression in PC tissues.

## Disscusion

PC is a highly aggressive malignancy with a poor prognosis. Despite significant advancements in treatment, patient survival rates remain low.^30^^,^^31^ The complex tumor microenvironment and its heterogeneity pose substantial challenges for effective therapy.^31^ Although immunotherapy has achieved breakthroughs in various cancers, the unique biological characteristics and immune microenvironment of PC limit its efficacy.^32^, ^33^, ^34^ As a result, the exploration of novel targets and combination treatment strategies is a crucial research priority.^35^ This study, integrating the TCGA and GTEx databases, employed differential expression analysis, Weighted Gene Co-expression Network Analysis (WGCNA), and Mendelian randomization (MR) analysis to reveal for the first time the crucial role of CLIC3 and MST1R in the immune mechanisms of PC. These genes are closely linked to the initiation, progression, and prognosis of PC and hold potential as drug targets with significant clinical relevance.

Despite multiple corrections for false positives using DESeq2, edgeR, limma, and the Wilcoxon rank-sum test to ensure result robustness,^36^ the study integrated these methods to comprehensively analyze differential expression. WGCNA analysis further confirmed the association of these genes with the biological processes and networks of PC, providing crucial insights into their molecular mechanisms.^37^ Single-factor COX and MR analyses indicated that CLIC3 and MST1R not only affect survival prognosis in PC patients but may also promote tumor progression, with a potential causal relationship between them.

Previous research has shown that smoking and a history of chronic pancreatitis are major risk factors for the onset of PC, and alcohol consumption may also contribute to the increased risk of PC through its link to chronic pancreatitis.^38^, ^39^, ^40^ In this study, patients with high expression levels of CLIC3 and MST1R were found to have a history of smoking and chronic pancreatitis, suggesting that these molecules may serve as potential molecular biomarkers for identifying individuals at high risk for PC. Moreover, among patients with alcohol consumption and more than 20 lymph nodes around the tumor, CLIC3 expression was significantly upregulated, indicating its involvement in tumor cell lymphatic metastasis and its potential as a therapeutic target to prevent PC lymphatic spread.

Immune infiltration analysis revealed that in the high expression groups of CLIC3 and MST1R, there was a higher infiltration level of M0 macrophages, while the infiltration of CD4+ T-cells, CD8+ T-cells, and B-cells was reduced. M0 macrophages play a pivotal role in the tumor immune microenvironment and can polarize into M1 or M2 phenotypes under different stimuli. M1 macrophages typically inhibit tumor progression in the early stages through anti-inflammatory actions, while M2 macrophages promote progression during tumor development.^41^ In the PC microenvironment, the infiltration of inflammatory cells may facilitate the polarization of M2 macrophages, thereby inhibiting the antitumor functions of CD8+ T-cells.^42^ Collectively, our findings indicate that high CLIC3 and MST1R expression is linked to a macrophage-dominant immune landscape accompanied by reduced cytotoxic T-cell abundance in pancreatic cancer. While these observations are derived from computational deconvolution and correlation analyses, they align with prior evidence connecting macrophage-rich microenvironments to impaired antitumor T-cell responses. Together, these results highlight a potential role for CLIC3 and MST1R in shaping the immunosuppressive tumor microenvironment, warranting further functional investigation to elucidate their mechanistic contributions.

Preliminary GSEA enrichment analysis suggests that CLIC3 and MST1R may participate in key pathways and cellular processes implicated in PC. Although this study primarily relies on bioinformatics analysis, existing literature provides functional evidence supporting the pivotal roles of CLIC3 and MST1R in tumor progression and immune regulation. Specifically, studies have shown that MST1R accelerates pancreatic cancer progression by promoting the infiltration of immunosuppressive macrophages, thereby remodeling the tumor microenvironment.^18^^,^^43^ While direct functional studies of CLIC3 in PC are limited, research in other malignancies has demonstrated that CLIC3 promotes tumor progression via modulation of key signaling pathways such as MAPK/ERK.^44^^,^^45^

To explore potential mechanistic links underlying the observed associations, the authors further examined pathway enrichment and gene co-expression patterns derived from our own transcriptomic data. Enrichment analyses revealed that genes co-expressed with CLIC3 and MST1R were significantly involved in pathways related to autophagy regulation, chemokine signaling, and immune-related processes. Although canonical PI3K/AKT or MAPK pathways were not uniformly enriched as top-ranked pathways, several functionally related downstream signaling modules were identified, suggesting possible indirect involvement of these axes. Based on these findings, the authors hypothesize that CLIC3 and MST1R may be functionally linked to PI3K/AKT and MAPK-related signaling networks, as suggested by prior studies in other malignancies, contributing to the development of chemotherapy resistance in PC. Furthermore, these two targets may regulate interactions between immune cells and tumor cells in the tumor microenvironment, ultimately forming a multidimensional pro-tumor network. This hypothesis warrants further experimental validation.

The single-gene enrichment analysis revealed that CLIC3 and MST1R play a critical role in the immune microenvironment by promoting autophagy regulation and inhibiting chemokine signaling pathways. Previous research has shown that in mice, knocking out the autophagy gene ATG5 results in an increase in M1 macrophages and a decrease in M2 macrophages, leading to acute liver damage and inflammation.^46^ Additionally, Fang et al.^47^ demonstrated that puerarin modulates the autophagy process through the ULK1-PAI-1 signaling pathway, affecting macrophage polarization by increasing the proportion of M2 macrophages, thereby reducing inflammation and exerting a protective effect against non-alcoholic steatohepatitis. In the context of PC, exosomes can promote macrophage polarization towards the M2 phenotype, enhancing tumor proliferation, migration, and invasion, and this process is closely associated with glycolysis and pyruvate metabolism.^48^^,^^49^ Therefore, integrating the findings from this study with previous research, it is reasonable to suggest that CLIC3 and MST1R may regulate macrophage proliferation (M2 polarization) through autophagy, thereby inhibiting CD8+ T-cell generation and facilitating the development and progression of PC. However, this mechanism requires further experimental validation.

This study stratified PC based on CLIC3 and MST1R expression, identifying an immunosuppressive microenvironment in Cluster 2 (characterized by increased M0 macrophages and decreased CD8⁺ T-cells). This milieu may weaken antitumor immunity, accelerate tumor progression, and contribute to poorer survival outcomes. These findings suggest that CLIC3 and MST1R may play critical roles in modulating the tumor immune microenvironment and driving PC heterogeneity. Although CIBERSORT has been widely employed for immune cell deconvolution, its precision in resolving macrophage subtypes, particularly within the complex microenvironment of pancreatic cancer, remains subject to ongoing debate. Consequently, the macrophage-associated findings presented herein should be interpreted with appropriate caution. Future studies leveraging single-cell transcriptomics or spatial immunophenotyping are necessary to substantiate and refine these observations. In the field of immunotherapy, the authors observed that in the high expression groups of CLIC3 and MST1R, there was an elevation in the expression of immune checkpoint molecules CD276 (B7-H3) and NT5E (CD73), with an associated increase in Tumor Mutational Burden (TMB), primarily due to KRAS and TP53 mutations. This finding may indicate a major immune evasion mechanism in PC. CD276, as an immune checkpoint molecule, is often closely linked to tumor immune escape and holds potential value in the prognosis and early diagnosis of PC.^50^ Miller et al.^51^ demonstrated that high CD276 expression in PC is closely associated with mutations in oncogenes such as KRAS and TP53, providing important insights into the immune evasion mechanisms and treatment responses in PC. Thus, CD276 may represent a promising immunotherapy target for patients with high expression of CLIC3 and MST1R in PC.

The co-localization analysis further reinforced the robustness of the Mendelian Randomization (MR) findings and highlighted the therapeutic potential of CLIC3 and MST1R as drug targets. It should be noted that the colocalization analysis relies on predefined prior probability assumptions, which may influence posterior estimates. Although commonly used parameter settings were applied in this study, future sensitivity analyses across a range of priors would further strengthen the robustness of these findings. In molecular docking simulations, Cabozantinib exhibited the strongest hydrogen bond binding affinity with MST1R. IBMX and HCtoxin also demonstrated high binding stability with CLIC3, although current literature regarding their interactions with CLIC3 remains limited. Additionally, Ampicillin showed relatively stable hydrogen bond interactions with CLIC3. The Phase III CABINET trial confirmed that Cabozantinib significantly prolonged progression-free survival in patients with pancreatic Neuroendocrine Tumors (pNET), offering a novel treatment option for advanced Neuroendocrine Tumors (NETs).^52^ Chandra et al.^53^ reported that Ampicillin could inhibit tumor growth by modulating the dysbiosis of pancreatic cancer-associated microbiota, indicating its potential in microbiota-targeted therapy. Increasing evidence suggests that tumor-associated microbes play a pivotal role in cancer progression.^54^ These findings open new avenues for the development of drug targets and precision therapies in pancreatic cancer. Despite the promising results obtained from molecular docking simulations, the authors acknowledge that these findings remain predictive in nature and lack experimental binding affinity validation. Techniques such as surface plasmon resonance or isothermal titration calorimetry would provide direct evidence for the physical interaction between the candidate molecules and target proteins. Due to current resource and technical limitations, such validation could not be performed in this study. Future investigations employing rigorous biochemical and biophysical assays are warranted to substantiate the computational predictions and further clarify the therapeutic potential of the identified compounds.

The apparently contradictory roles of CLIC3 reported across different tumor types highlight its strong context-dependent biological function. In gastric cancer, CLIC3 has been suggested to exert tumor-suppressive effects, potentially through epithelial homeostasis-related mechanisms, whereas in bladder cancer and other malignancies, elevated CLIC3 expression has been associated with aggressive phenotypes.^12^ Pancreatic cancer is characterized by a uniquely dense stromal architecture and a profoundly immunosuppressive microenvironment. In this context, the present findings suggest that CLIC3 overexpression is associated with a macrophage-dominant immune infiltration pattern and reduced cytotoxic T-cell abundance, indicating that its functional role may be reshaped by tumor-specific microenvironmental constraints. These observations underscore the importance of considering tissue context and immune landscape when interpreting the biological role of CLIC3 across cancers. In pancreatic cancer, the mechanism of CLIC3 differs from that in gastric cancer. This study focuses on its role in the immune microenvironment of pancreatic cancer and suggests that it may influence immune evasion and prognosis. Therefore, despite the existing controversies in gastric cancer, the potential function of CLIC3 in pancreatic cancer still requires further validation and may provide new perspectives for cancer immunotherapy.

This study systematically integrated multi-omics data to elucidate for the first time the pathogenic mechanism and clinical significance of CLIC3 and MST1R in PC. The results revealed that CLIC3 and MST1R significantly influence the tumor microenvironment by regulating autophagy and chemokine signaling, particularly affecting macrophages and CD8+ T-cells, and are closely associated with the immune checkpoint molecules CD276 and NT5E, highlighting their critical role in immune escape in PC. Drug target analysis identified stable binding of Cabozantinib with MST1R and the potential of Ampicillin in microbiome regulation, offering new avenues for early diagnosis and targeted therapy in PC. Importantly, the proposed mechanistic links between CLIC3/MST1R and these signaling pathways are primarily hypothesis-generating and are not intended to imply direct molecular regulation, which will require targeted functional validation in future experimental studies. However, this study has limitations, including the lack of in vitro and in vivo validation, sample heterogeneity, and challenges in clinical translation. Future efforts should validate the involvement of CLIC3 and MST1R in related pathways through cell and animal experiments and investigate their potential in combination immunotherapy through multicenter clinical studies to drive breakthroughs in PC treatment.

## Conclusions

In conclusion, the elevated expression of CLIC3 and MST1R in PC is strongly associated with tumorigenesis, disease progression, and poor patient prognosis. High expression levels reshape the immune microenvironment, marked by an increased abundance of M0 macrophages and reduced CD8+ T-cells and CD4+ T memory subsets, thereby facilitating tumor progression through autophagy and chemokine signaling pathways. Moreover, their expression correlates with elevated levels of immune checkpoint molecules (CD276 and NT5E) and higher KRAS and TP53 mutation burdens, suggesting a role in driving PC progression via immune evasion. Molecular docking studies further reveal that Cabozantinib is a potential therapeutic agent targeting MST1R, while Ampicillin shows promise in microbial-targeted therapy, offering innovative strategies for advancing immunotherapy and targeted therapy approaches.

## Data availability

The dataset used for the analyses in this study is available to qualified researchers upon request. Please email the co-first author, Changjun Dong at (202210510021014@ctgu.edu.cn) or the corresponding author, Xianlin Zhang, Ph.D., at (xianlin.zhang@163.com).

## Ethical approval

This study was reviewed and approved by the Ethics Committee of the Affiliated Renhe Hospital of China Three Gorges University (Approval n° 2023–20). All procedures were performed in accordance with the relevant guidelines and regulations. Informed consent was obtained from all participants or their legal guardians prior to participation.

## Authors’ contributions

Changjun Dong: Writing-review & editing; writing-original draft; visualization; supervision; project administration; methodology; formal analysis; data curation; conceptualization. Jing Guan: Writing-review & editing; writing-original draft; project administration; data curation. Linhuan Dong: Writing-original draft; formal analysis. Yunlin Yu: Software; data curation. Xiangwei Zhang: Software; data curation. Zheng Li: Funding support. Xianlin Zhang: Software; Formal analysis; data curation; funding support. Chunlin Mo: Software, Formal analysis; data curation.Chunlin Mo contributed equally with the corresponding author, Xianlin Zhang.

## Funding

This work was supported by the 10.13039/501100003819Natural Science Foundation of Hubei Province (grant number 2022CF328).

## Declaration of competing interest

The authors declare that they have no known competing financial interests or personal relationships that could have appeared to influence the work reported in this paper.

## Appendix Supplementary materials

Image, application 1

Image, application 2

Image, application 3

## Footnotes

Supplementary material associated with this article can be found, in the online version, at doi:10.1016/j.clinsp.2026.100952.
