# Supplementary material for: Investigation of molecular immune regulation mechanisms and therapeutic target prediction in pancreatic cancer based on bioinformatics and genetics
Source: Clinics (Sao Paulo). 2026 Apr 18;81:100952. doi: 10.1016/j.clinsp.2026.100952 (PMC13098448; doi:10.1016/j.clinsp.2026.100952)
Supplement: Supplementary file 3 [file mmc3.doc]

**Supplementary Information file.:**

**Investigation of Molecular Immune Regulation Mechanisms and Therapeutic Target Prediction in Pancreatic Cancer Based on Bioinformatics and Genetics**

Changjun Dong^1,2,^**^+^**,Jing Guan^1,^**^+^**,Linhuan Dong^1^,Yunlin Yu^1^,Xiangwei Zhang^1^，Xianlin Zhang^1,2*^ Zheng Li^1,2*^

***Co-first authors：***Changjun Dong and Jing Guan are co-frst authors and contributed equally to this work.

**1** Department of General surgery, Afliated Renhe Hospital of China Three Gorges University,Yichang 443000, China

**1** Hubei Key Laboratory of Tumor Microenvironment and Immunotherapy, China Three Gorges University, Yichang 443002, China

***Corresponding author：***Xianlin Zhang* and Zheng Li*

**Supplementary Figues（1-6）：**

**Supplementary Figue S1** Batch effects in samples from the TCGA and GTEx databases before merging (A) and after correction (B). Intersection of downregulated genes obtained via four differential expression analysis methods (C) and upregulated genes (D).

**Supplementary Figue S2**. Box plots illustrating the differences in CLIC3 expression across subgroups classified by tumor grade (A), clinical stage (B), history of alcohol consumption (C), history of smoking (D), history of chronic pancreatitis (E), and the number of peritumoral lymph nodes (F). Similarly, differences in MST1R expression are shown in subgroups classified by tumor grade (E) and chronic pancreatitis history (G).

**Supplementary Figue S3**. Analysis of the correlation between CLIC3 (A) and MST1R (B) with 12 common immune checkpoints in PC.

**Supplementary Figue S4** Colocalization analysis of CLIC3 (A) and MST1R (B); correlation analysis between CLIC3 and MST1R (C); protein-protein interaction (PPI) network analysis (D).

**Supplementary Figue S5** Correlation analysis between the expression levels of CLIC3 (A) and MST1R (B) and tumor mutation burden (TMB); scatter plot depicting mutations per million bases in PC tissues (C); visualization of TMB comparison between high and low expression groups of CLIC3 (D-E) and MST1R (F-G).

**Supplementary Figue S6**. Molecular docking analysis of predicted drugs with CLIC3 and MST1R targets. Docking results for ampicillin, 3-isobutyl-1-methylxanthine, and HC cyclopeptide toxin with CLIC3 (A-B, F), and for cabozantinib, bosutinib, and erlotinib with MST1R (C-E).
